# Frailty and mortality are not influenced by mitochondrial DNA haplotypes in the very old^☆^

**DOI:** 10.1016/j.neurobiolaging.2013.04.001

**Published:** 2013-12

**Authors:** Joanna Collerton, Deepthi Ashok, Carmen Martin-Ruiz, Angela Pyle, Gavin Hudson, Mohammad Yadegarfar, Karen Davies, Carol Jagger, Thomas von Zglinicki, Thomas B.L. Kirkwood, Patrick F. Chinnery

**Affiliations:** a Institute for Ageing and Health, Newcastle University, Campus for Ageing and Vitality, Newcastle upon Tyne NE4 5PL, UK; b Wellcome Trust Centre for Mitochondrial Research, Institute of Genetic Medicine, Newcastle University, Newcastle upon Tyne NE1 4LP, UK

**Keywords:** Mitochondrial DNA, Aging, Frailty, Genetics

## Abstract

Inherited genetic variation of mitochondrial DNA (mtDNA) could account for the missing heritability of human longevity and healthy aging. Here, we show no robust association between common genetic variants of mtDNA and frailty (an “unhealthy aging” phenotype) or mortality in 700, more than 85-year-old, participants of the Newcastle 85+ study. Conflicting data from different populations underscore our conclusion that there is currently no compelling link between inherited mtDNA variants and aging.

## 1 Introduction

Human longevity shows heritability of ∼25%, but large-scale, nuclear, genome-wide association studies have not yet clearly established all the responsible genes (Beekman et al., 2013; Kirkwood et al., 2011). In addition to nuclear genes, there may also be contributions from the maternally inherited, extranuclear, mitochondrial genome (mitochondrial DNA [mtDNA]). mtDNA codes for 13 respiratory chain proteins that are essential for the production of adenosine triphosphate, which is required for all active intracellular processes. There is an emerging evidence that mitochondrial dysfunction plays a key role in cellular aging, and the accumulation of somatic mutations of mtDNA is associated with an aging phenotype in mice and humans (Schon et al., 2012). It is, therefore, of great interest that different polymorphic variants of mtDNA appear to be enriched in cohorts of healthy older individuals, raising the possibility that our mitochondrial genome may influence how we age and how long we live. However, although several studies support this hypothesis (Niemi et al., 2003, 2005; Yang et al., 2012), there are conflicting findings and a lack of consistency (Courtenay et al., 2012; De Benedictis et al., 1999; Feng et al., 2011; Finnila et al., 2000; Ivanova et al., 1998; Ross et al., 2001; Tanaka et al., 1998, 2000). Moreover, there have been no studies of mtDNA and healthy aging phenotypes.

In an attempt to resolve these issues, we carried out a comprehensive study of mtDNA haplogroups in the Newcastle 85+ study. Ten mtDNA haplogroup markers effectively tag the most common subgroups of mtDNA found in 95% of the local population. The Newcastle 85+ study provides a unique opportunity to study the effects of these haplogroups both on survival up to and beyond age 85 and on frailty, an “unhealthy aging” phenotype, in a representative population-based cohort of the very old.

## 2 Experimental procedures

### 2.1 Study cohort

The Newcastle 85+ study has been reported and includes a sociodemographically representative 1921 birth cohort recruited at age ∼85 through general practice patient lists (*n* = 845) (Collerton et al., 2009). An assessment of frailty was performed at baseline using 2 robust and validated measures: the Rockwood frailty index (RFI) (Rockwood and Mitnitski, 2007) and the Fried frailty status (FFS) (Fried et al., 2009), as described (Collerton et al., 2012). RFI was available for 811 (96.0%) of the cohort and FFS for 552 (65.3%). The cohort has been followed for mortality from baseline assessment (June 2006 to Sept 2007) until April 30, 2012.

### 2.2 Molecular genetic analysis

mtDNA haplogroups were determined using a stepwise algorithm (Torroni et al., 1996) by primer extension of multiplex polymerase chain reaction products with the detection of the allele-specific extension products by matrix-associated laser desorption/ionization time of flight (Sequenom MassARRAY, San Diego, CA, USA). DNA was available for 752 participants; 52 cases were excluded from the analysis because of either heteroplasmic status (*n* = 5) or inability to detect haplogroup/low-quality DNA (*n* = 47), leaving 700 with valid mitochondrial haplogroup data.

### 2.3 Statistical analysis

The frequency of mtDNA haplogroups in the incident Newcastle 85+ cohort (*n* = 700) was compared with 3 ethnically matched population control data sets representing different ages using chi-squared analysis with pairwise comparisons of samples for each haplogroup: (1) a local birth cohort (*n* = 344, neonatal cord blood samples), North Cumbria Community Genetics Project (Elliott et al., 2008); (2) a national mid-age cohort, the 1958 Medical Research Council cohort (*n* = 2889, 52% male), which has previously been shown to be representative of control subjects in our region (Chinnery et al., 2007); and (3) a local cohort of healthy older subjects (*n* = 93, 35% male; mean age 69, standard deviation = 8.5).

Mitochondrial haplogroup data were available for 85.8% (696/811) of those with RFI available and 91.4% (477/552) of those with FFS available. These were the samples used in the principal analyses. Linear regression was used to determine the relationship between mtDNA haplogroups and RFI, and ordinal logistic regression was used to determine the relationship between mtDNA haplogroups and FFS, both before and after controlling for sex, years of education, and smoking status. A count of chronic diseases was used as an additional control for the FFS. The relationship between mtDNA haplogroups and survival was determined by Cox proportional hazards analysis, both before and after controlling for sex, total cholesterol, body mass index, hypertension, diabetes, ethnicity, and smoking status. The median follow-up period was 58 months during which 336 deaths occurred.

## 3 Results

The overall frequency distribution of mtDNA haplogroups in the incident Newcastle 85+ cohort was compared with the birth cohort (*p* = 344), mid-age cohort (*p* = 2889), and old-age cohort (*p* = 93) (Table 1). The only significant difference in distributions was for the local older cohort that differed from each of the other cohorts in the “other” haplogroup category only.

There was no significant association between mtDNA haplogroups and frailty (RFI or FFS) before and after controlling for the potential confounding variables (Tables 2 and 3). Although we observed an association between haplogroup X and increased mortality (*p* = 0.025), this was not apparent after controlling for total cholesterol, body mass index, hypertension, diabetes, ethnicity, and smoking status (Table 4). Likewise, the trend toward reduced mortality associated with haplogroup K (*p* = 0.041), which remained after controlling for other variables (*p* = 0.023), did not withstand correction for the multiple haplogroups under investigation.

## 4 Discussion

We found no robust evidence of an association between mtDNA haplogroups and either frailty or survival beyond age 85 or any informative biomarker of aging (Martin-Ruiz et al., 2011), and the overall distribution of mtDNA haplogroups closely resembled ethnically matched cohorts from 3 different younger age groups. Our findings do not support a role for mtDNA in promoting healthy aging or longevity. The absence of an age-associated stratification of mtDNA haplogroups is in agreement with previous findings in large European cohorts (Benn et al., 2008; Dato et al., 2004). On the other hand, our findings contrast with the results of several smaller European (De Benedictis et al., 1999; Dominguez-Garrido et al., 2009; Ivanova et al., 1998; Niemi et al., 2003; Ross et al., 2001) and Far-Eastern studies (Feng et al., 2011; Zhang et al., 2003). Although it is conceivable that these geographic differences reflect different environmental constraints, or ethnic differences in the nuclear genetic background, it is perhaps more likely that the relatively small size of these study groups led to false-positive associations because mtDNA haplogroup studies are particularly sensitive to population stratification. In keeping with this, none of the previously reported positive findings have been replicated directly, and it is not clear why a particular haplogroup would be associated with longevity in one context and not the other. Likewise, we were unable to replicate previous findings of a gender-specific association with aging. If present, such an association would be difficult to explain mechanistically.

Although we cannot exclude the possibility that a larger study cohort would reveal an association between mtDNA and longevity and/or healthy aging phenotypes, our findings of a lack of an association with 2 sensitive and reliable measures of frailty and no direct evidence of an effect on survival suggest that any contribution from mtDNA would be modest at best. Our results, therefore, turn the spotlight away from mtDNA back to the nuclear genome, in the search for genes predisposing to longevity and healthy aging.

## Disclosure statement

The authors report no conflicts of interest.

The data contained in the manuscript being submitted have not been previously published, have not been submitted elsewhere, and will not be submitted elsewhere while under consideration at *Neurobiology of Aging*. All authors have reviewed the contents of the manuscript being submitted, approved of its contents, and validated the accuracy of the data.

Ethical approval for the study is in place.

## Figures and Tables

**Table 1:** Comparison of haplogroup frequencies across cohorts, percent (*n*)

Mitochondrial haplogroup	Newcastle 85+ study age 85.5 y (SD 0.4), *n* = 700	Local older cohort age 68.9 y (SD 8.5), *n* = 93	1958 MRC birth cohort, *n* = 2889	Local birth cohort neonates, *n* = 344
H	46.0 (322)	36.6 (34)	44.2 (1278)	39.8 (137)
V	3.6 (25)	3.2 (3)	3.0 (87)	3.5 (12)
J	11.0 (77)	8.6 (8)	11.6 (336)	9.9 (34)
T	10.3 (72)	3.2 (3)	10.0 (289)	13.1 (45)
U	15.4 (108)	15.1 (14)	12.9 (374)	15.1 (52)
K	7.3 (51)	7.5 (7)	9.3 (269)	7.8 (27)
Other (W, X, I, M classified here)	6.4 (45)	25.8 (24)	8.9 (256)	10.8 (37)

Key: SD, standard deviation; MRC, Medical Research Council.

**Table 2:** RFI and mitochondrial haplogroup—regression coefficients (unstandardized) for square root–transformed RFI by mitochondrial haplogroup^a^

Haplogroup	Model 1, unstandardized regression coefficient (95% confidence interval)	Model 1, *p* value	Model 2, unstandardized regression coefficient (95% confidence interval)	Model 2, *p* value	Model 3, unstandardized regression coefficient (95% confidence interval)	Model 3, *p* value
H	0.015 (−0.003 to 0.034)	0.101	0.017 (−0.001 to 0.036)	0.065	0.013 (−0.006 to 0.031)	0.175
Non-H	Reference		Reference		Reference	
T	−0.006 (−0.037 to 0.024)	0.684	−0.009 (−0.040 to 0.021)	0.536	−0.007 (−0.036 to 0.023)	0.663
Non-T	Reference		Reference		Reference	
J	−0.016 (−0.045 to 0.014)	0.300	−0.014 (−0.043 to 0.015)	0.338	−0.011 (−0.040 to 0.018)	0.455
Non-J	Reference		Reference		Reference	
U	−0.009 (−0.035 to 0.016)	0.481	−0.009 (−0.034 to 0.016)	0.488	−0.008 (−0.033 to 0.017)	0.526
Non-U	Reference		Reference		Reference	
K	−0.033 (−0.068 to 0.003)	0.069	−0.033 (−0.068 to 0.002)	0.064	−0.029 (−0.064 to 0.007)	0.112
Non-K	Reference		Reference		Reference	
X	0.060 (−0.008 to 0.128)	0.083	0.057 (−0.010 to 0.124)	0.098	0.053 (−0.014 to 0.120)	0.122
Non-X	Reference		Reference		Reference	
W	0.015 (−0.040 to 0.071)	0.585	0.012 (−0.043 to 0.066)	0.675	0.018 (−0.037 to 0.072)	0.523
Non-W	Reference		Reference		Reference	
I	0.052 (−0.022 to 0.126)	0.170	0.047 (−0.026 to 0.120)	0.206	0.051 (−0.022 to 0.123)	0.169
Non-I	Reference		Reference		Reference	
V	−0.019 (−0.068 to 0.031)	0.461	−0.020 (−0.069 to 0.029)	0.424	−0.020 (−0.069 to 0.028)	0.410
Non-V	Reference		Reference		Reference	

Key: RFI, Rockwood frailty index.

a Linear regression models were fitted with RFI (square root transformed to give adequate model fit) as the dependent variable and mitochondrial haplogroup as the independent variable. Seven binary variables were created of “in haplogroup H” versus “not in haplogroup H” type, and 7 models were run entering each binary variable separately. Model 1 is unadjusted, model 2 adjusted for sex, and model 3 adjusted for sex, education, and smoking status.

**Table 3:** FFS and mitochondrial haplogroup—odds ratio (95% confidence interval) of being in a frailer Fried category (pre-frail versus robust or frail versus pre-frail) by mitochondrial haplogroup^a^

Haplogroup	Model 1, odds ratio (95% confidence interval)	Model 1, *p* value	Model 2, odds ratio (95% confidence interval)	Model 2, *p* value	Model 3, odds ratio (95% confidence interval)	Model 3, *p* value
H	1.05 (0.73–1.50)	0.790	1.10 (0.77–1.57)	0.608	1.04 (0.71–1.53)	0.832
Non-H	Reference		Reference		Reference	
T	1.10 (0.61–1.96)	0.752	0.93 (0.52–1.68)	0.815	0.80 (0.43–1.51)	0.498
Non-T	Reference		Reference		Reference	
J	0.72 (0.41–1.27)	0.262	0.74 (0.42–1.31)	0.305	0.99 (0.54–1.81)	0.968
Non-J	Reference		Reference		Reference	
U	0.95 (0.59–1.52)	0.816	0.95 (0.59–1.52)	0.820	0.85 (0.51–1.41)	0.526
Non-U	Reference		Reference		Reference	
K	0.66 (0.33–1.32)	0.248	0.70 (0.35–1.37)	0.293	0.79 (0.38–1.64)	0.532
Non-K	Reference		Reference		Reference	
X	4.45 (0.91–21.71)	0.065	4.32 (0.87–21.52)	0.074	3.64 (0.68–19.61)	0.132
Non-X	Reference		Reference		Reference	
W	2.58 (0.88–7.57)	0.084	2.30 (0.78–6.81)	0.131	2.90 (0.89–9.49)	0.078
Non-W	Reference		Reference		Reference	
I	0.95 (0.10–9.10)	0.967	0.89 (0.09–8.53)	0.918	1.14 (0.11–11.78)	0.913
Non-I	Reference		Reference		Reference	
V	1.20 (0.43–3.32)	0.729	1.29 (0.46–3.58)	0.630	1.20 (0.38–3.71)	0.757
Non-V	Reference		Reference		Reference	

Key: FFS, Fried frailty status.

a Ordinal logistic regression models were fitted with Fried status as the dependent variable and mitochondrial haplogroup as the independent variable. Seven binary variables were created of “in haplogroup H” versus “not in haplogroup H” type, and 7 models were run entering each binary variable separately. Model 1 is unadjusted, model 2 adjusted for sex only, and model 3 adjusted for sex, years of education, smoking status, and count of chronic diseases.

**Table 4:** Hazard ratios for mortality by mitochondrial haplogroup^a^

Haplogroup	Model 1, hazard ratio (95% confidence interval)	Model 1, *p* value	Model 2, hazard ratio (95% confidence interval)	Model 2, *p* value
H	1.10 (0.89–1.36)	0.380	1.04 (0.82–1.31)	0.748
Non-H	Reference		Reference	
T	1.00 (0.70–1.43)	0.988	1.04 (0.72–1.50)	0.852
Non-T	Reference		Reference	
J	0.92 (0.65–1.30)	0.625	0.95 (0.66–1.36)	0.762
Non-J	Reference		Reference	
U	0.97 (0.72–1.31)	0.841	1.13 (0.83–1.55)	0.425
Non-U	Reference		Reference	
K	0.61 (0.38–0.98)	**0.041**	0.52 (0.30–0.91)	**0.023**
Non-K	Reference		Reference	
X	2.05 (1.09–3.86)	**0.025**	1.91 (0.96–3.83)	0.066
Non-X	Reference		Reference	
W	0.85 (0.42–1.72)	0.652	0.90 (0.42–1.92)	0.790
Non-W	Reference		Reference	
I	0.99 (0.41–2.38)	0.973	0.71 (0.26–1.93)	0.506
Non-I	Reference		Reference	
V	1.28 (0.75–2.19)	0.360	1.22 (0.68–2.19)	0.497
Non-V	Reference		Reference	

Bold text indicates *p* < 0.05.

a Seven binary variables were created of “in haplogroup H” versus “not in haplogroup H” type, and 7 models were run entering each binary variable separately. Model 1 is unadjusted and model 2 is adjusted for sex, ethnicity, total cholesterol, body mass index, hypertension, diabetes, and smoking.
